# A Randomized, Double-Blind, Parallel-Controlled Trial: Addressing Kinesiophobia in Post-Meniscoplasty Patients Through Progressive Muscle Relaxation Training and Acupressure

**DOI:** 10.1155/prm/1270985

**Published:** 2025-03-31

**Authors:** Rui Xu, Junfang Miao, Yingxia Gong, Hongan Jia, Huijuan Wu, Weizhao Wang, Huijuan Wang, Mengmeng Dong, Ying Zhang

**Affiliations:** ^1^School of Nursing, Gansu University of Chinese Medicine, Lanzhou, China; ^2^Nursing Department, The First People's Hospital of Baiyin, Baiyin, China; ^3^Department of Orthopedics, Gansu Provincial People's Hospital, Lanzhou, China; ^4^Nursing Department, Xijing Hospital of Air Force Military Medical University, Xi'an, China; ^5^Nursing Department, Stomatology Hospital of Lanzhou University, Lanzhou, China; ^6^School of Nursing, Wuwei Vocational College, Wuwei, China

**Keywords:** acupressure, kinesiophobia, meniscoplasty, progressive muscle relaxation training

## Abstract

**Background:** The increasing prevalence of kinesiophobia, or the fear of movement, among patients following meniscectomy has necessitated the exploration of effective rehabilitative interventions. Traditional methods of rehabilitation often do not adequately address the psychological components of recovery, leading to prolonged recovery times and decreased quality of life.

**Objective:** The objective of this study is to explore the effectiveness of psychological and traditional Chinese medical techniques, including progressive muscle relaxation training (PMRT) and acupressure, in treating kinesiophobia among patients after meniscus surgery.

**Methods:** This randomized clinical trial commenced in December 2021 at the Sports Medicine Department of a hospital in Gansu Province and concluded in February 2023. Seventy hospital inpatients with movement disorders who had undergone meniscus shaping surgery participated in the study (experimental Group 35 people, control Group 35 people). The control group received standard care. In addition to receiving routine care, the experimental group underwent an additional 30 min of PMRT and 5–10 min of acupressure. The kinesiophobia scores and pain scores were assessed using the Tampa Scale for Kinesiophobia (TSK) and Visual Analogue Scale (VAS) before the intervention, the first day after the intervention, the fifth day after the intervention, and on the day of discharge. On the day of discharge, the Knee Society Score (KSS) was used to assess the knee joint function of the patients. Statistical analysis was performed using repeated measures ANOVA.

**Results:** The study included 70 kinesiophobia patients following meniscoplasty, equally split between the experimental and control groups. Attrition resulted in 3 experimental group withdrawals and 2 from the control group, leaving 65 for the final analysis (32 experimental, 33 control). The average age of the patients was (67.03 ± 8.26) years, with an average BMI of (25.09 ± 2.88) kg/cm^2^. Females accounted for 66.10% of the participants. There were no statistically significant differences between the two groups in terms of their preintervention TSK scores, VAS scores, and other baseline data (*p* > 0.05).There were no statistically significant differences in the kinesiophobia scores (TSK scores) and pain scores (VAS scores) between the experimental group and the routine care group both before the intervention and on the first day after the intervention (*p* > 0.05). However, the fifth-day and discharge assessments revealed significant score improvements in the experimental group (*p* < 0.05), along with KSS scores indicating enhanced knee joint function compared to controls (*p* < 0.05).

**Conclusions:** PMRT combined with acupressure effectively decreases kinesiophobia levels post-meniscoplasty, mitigates pain, fosters early functional exercise participation, and promotes knee joint function recovery.

**Trial Registration:** ClinicalTrials.gov identifier: NCT06409715

## 1. Introduction

The meniscus, a crucial component of the knee joint, plays a vital role in shock absorption, lubrication, and joint stability [1]. Meniscal tears are common knee injuries with an annual incidence of approximately 0.6–0.7 cases per 1000 person-years in the general population. The incidence of meniscal tears is higher in sports-related knee injuries [2]. A review of data on meniscus surgery in the United States from 2010 to 2020 revealed a decrease in the overall incidence of meniscus surgery, with the most significant decrease in the incidence of partial meniscectomy. In contrast, there was an increase in the incidence of meniscal repair and transplantation procedures [3]. These injuries can lead to significant degenerative changes in the articular cartilage, increasing the risk of developing knee osteoarthritis [4]. Given the limited intrinsic capacity of the meniscus for self-repair due to its poor blood supply [5], early intervention and preservation of meniscal integrity are essential.

Arthroscopic surgery is the standard treatment for meniscal injuries. Despite being minimally invasive, patients often experience postoperative discomfort, including pain, swelling, and reduced range of motion. A significant psychological concern that may arise postsurgery is kinesiophobia, which refers to an irrational fear of movement due to the apprehension of re-injury or pain [6]. This phenomenon often manifests after surgical intervention. A study in China found that among 123 patients diagnosed with meniscal injuries at Fujian Provincial Hospital, 60.16% had kinesiophobia [7]. The research also discovered that kinesiophobia was significantly associated with the severity of the injury, restricted joint movement, pain intensity, self-efficacy, and other functional parameters.

Recent literature has highlighted the importance of addressing kinesiophobia to improve rehabilitation outcomes. Various interventions, including cognitive-behavioral therapy and multidisciplinary approaches, have been shown to reduce symptoms of kinesiophobia and enhance the quality of life for affected individuals [8, 9]. However, these approaches often face challenges, such as high costs, the necessity for effective communication among healthcare providers, and the risk of inadvertently exacerbating patients' fear of movement [10]. Therefore, there is a pressing need for further research to refine and enhance intervention strategies that specifically target kinesiophobia in the context of rehabilitation.

In the context of post-meniscoplasty patients, kinesiophobia is a significant barrier to rehabilitation, often leading to reduced physical activity and decreased quality of life [11]. The use of modern psychological and rehabilitation techniques, such as virtual reality (VR) therapy and mobile applications, has also been explored to address kinesiophobia. For example, VR therapy has been shown to reduce kinesiophobia levels in patients with chronic musculoskeletal pain by creating controlled environments where patients can gradually acclimate to movements that induce fear [12]. Similarly, mobile applications and rehabilitation robotics have been used to enhance patient engagement and adherence to rehabilitation protocols, further supporting the management of kinesiophobia [13]. In addition to established strategies, complementary techniques such as progressive muscle relaxation training (PMRT) have shown promise in managing kinesiophobia. PMRT, which involves structured muscle contraction and relaxation, has been effective in reducing anxiety and pain, thereby alleviating stress responses in patients [14]. Furthermore, acupressure, rooted in traditional Chinese medicine, has been recognized for its therapeutic effects, including pain relief and improved recovery following musculoskeletal injuries [15, 16]. However, the effectiveness of these techniques in addressing kinesiophobia in post-meniscoplasty patients remains understudied.

This study aims to investigate the effectiveness of PMRT and acupressure in alleviating kinesiophobia in patients after meniscal surgery. We hypothesize that integrating these psychological and traditional Chinese medicinal techniques can enhance knee joint function rehabilitation, reduce pain, and alleviate fear, thus providing a culturally relevant and easily implementable approach to patient care.

By exploring the interplay between psychological interventions and traditional methods, this study seeks to provide valuable insights for both clinicians and researchers. For clinicians, incorporating PMRT and acupressure into rehabilitation programs may offer new therapeutic tools, particularly for patients whose psychological barriers are inadequately addressed by conventional methods. For patients and their families, discovering accessible options beyond standard rehabilitation practices can empower them in the recovery process, promoting better outcomes and overall well-being.

## 2. Methods

### 2.1. Study Design

This study utilized a randomized, double-blind, parallel-controlled trial design.

### 2.2. Study Population

This study received approval from the Ethics Committee of a tertiary class A hospital in Lanzhou ([2021] 59) [17]. The research period extended from December 2021 to February 2023. Subjects comprised patients who developed kinesiophobia following meniscus shaping surgery within the Sports Medicine Department of the aforementioned hospital in Lanzhou. Before the intervention, researchers explained the purpose, methods, and significance of the study to the patients, who then signed an informed consent form to ensure their voluntary participation. The patients were also informed of their right to participate and withdraw at any time.

#### 2.2.1. Inclusion Criteria

Inclusion criteria were as follows: (1) patients confirmed with meniscal damage as per established diagnostic guidelines and subjected to meniscus sculpting surgery; (2) individuals postmeniscus surgery exhibiting a Tampa Scale for Kinesiophobia (TSK) score exceeding 37 points [18]; (3) first-time patients receiving unilateral meniscal surgery; (4) exclusive employment of the meniscus sculpting surgical procedure; and (5) participation were entirely voluntary for all research participants.

#### 2.2.2. Exclusion Criteria

Exclusion criteria included: (1) patients with compromised consciousness or communicative impairments; (2) individuals with a history or presence of postsurgical lower limb thrombosis, resulting in activity limitation; (3) patients enduring deformities in the hip or ankle joints; and (4) those previously engaged in analogous research endeavors.

#### 2.2.3. Criteria for Withdrawal

Criteria for withdrawal incorporated: (1) patients opting out or becoming untraceable during therapeutic intervention and (2) individuals presenting serious medical conditions during treatment, impeding continued participation.

#### 2.2.4. Criteria for Elimination

Criteria for elimination encompassed: (1) subjects withdrawing or not reachable throughout the study timeline; (2) surgical patients subsequently transferring to alternative medical institutions; and (3) participants encountering unique circumstances during the study that prevents sustained involvement.

#### 2.2.5. Sample Size Calculation

The sample size calculation followed the two-sample means comparison formula outlined in “Statistics and Software Applications in Traditional Chinese Medicine” [19]. The formula is as follows:

(1) $$\begin{array}{c} n=\frac{2\left\{\left(\mu_{\alpha }+\mu_{\beta }\right)\right.*{\left.\sigma |\text{⁣}\right\}}^{2}}{\delta^{2}}. \end{array}$$

In this study, a two-tailed significance level (*α*) of 0.05 and a one-tailed significance level (*β*) of 0.1 were employed. The variables were defined as *δ* = 2.51 and *σ* = 4.23. Using statistical distributions, values for *μ*_*α*_ = 1.96 and *μ*_*β*_ = 1.282 were determined. Based on these values, the required sample size for each group was calculated to be approximately 30 participants.

To account for a potential 15% dropout rate, the total sample size was adjusted to 70 participants, with 35 participants in each group. This adjustment ensures sufficient statistical power (80%) even in the event of participant withdrawal or loss to follow-up.

In addition to dropout rates, we also considered potential sources of variability that could impact power, such as differences in baseline characteristics (e.g., age, gender, comorbidities). These factors were not explicitly incorporated into the sample size calculation but will be addressed during data analysis through appropriate statistical methods, such as stratification or regression models, to minimize their impact on the study's power.

By accounting for these factors, we aim to ensure that the study maintains adequate power to detect clinically meaningful differences between the experimental and control groups.

#### 2.2.6. Allocation Methodology

(1) Enumeration: The 70 patients selected for the study were sequentially numbered from 01 to 70. (2) Randomization: Randomization was carried out using Microsoft Excel's RAND function to generate a set of 70 random numbers. Starting from the second row and fourth column, the process continued sequentially to ensure an even distribution. In the event of duplicate numbers, they were replaced with new random selections. This ensured that each participant had an equal chance of being assigned to either the experimental or control group. (3) Envelope System: The 70 random numbers were inscribed on individual cards, each corresponding to a specific patient. These cards were then placed in sealed envelopes to ensure concealment of allocation. The first 35 patients were assigned to the experimental group, and the remaining 35 were assigned to the control group.

### 2.3. Intervention Protocols

The control group was administered standard care, whereas the experimental group received PMRT in conjunction with Acupressure, building upon the foundation of routine care. The above measures were implemented by several Masters of Traditional Chinese Medicine Nursing with extensive clinical experience.

#### 2.3.1. Standard Care for Control Group (See Appendix 1)

Following recommended protocols from “Arthroscopic Surgery and Sports Rehabilitation Nursing” [20], post-meniscectomy patient care primarily involves vital sign monitoring, positional support, dietary management, pain control, psychological support, functional training, and educational initiatives on health matters.

### 2.4. Experimental Group: Enhanced Care With PMRT and Acupressure

#### 2.4.1. PMRT (See Appendix 1)

Upon inclusion in the experimental arm of the study, participants were provided with a PMRT program sourced from the Chinese Medical Association Audiovisual Publishing House, containing 11 sequential steps. As per the regimen, patients were taught to induce and perceive tension and relaxation within the muscles. Each step involved sustaining muscular tension for 10 s, followed by relaxation for 5 s, repeating each step twice to facilitate postrelaxation comfort. PMRT commenced on postoperative day 3, barring any complications, and was scheduled following standard treatments twice daily, with each session lasting approximately 30 min [21].

#### 2.4.2. Acupressure Protocol

The patients rested briefly after PMRT before undergoing an Acupressure regimen implemented by the researchers. This process began with a 5–10-min application of kneading techniques around the knee complemented by quadricep pinching. Light kneading around the patella and its periphery followed. Progressive passive flexions and extensions of the knee were conducted, within the limits of the patients' pain thresholds. During the massage process, acupoint pressure was applied to Zusanli (S36), Sanyinjiao (SP6), and Yanglingquan (GB34), holding the pressure for 10 s upon eliciting sensations of soreness or numbness, reinforcing meridian flow. This protocol was cycled 3–5 times per acupoint, performed twice daily for roughly 5 minutes, and concluded with a palm percussion technique on the calf muscles to induce relaxation.

### 2.5. Outcome Measures

Assessments were conducted by trained, research-certified independent evaluators who were blinded to treatment conditions. The evaluators were not informed about the participants' group assignments and conducted the assessments in a manner that ensured the outcomes were evaluated independently of the interventions received by the participants. This process ensured that neither the participants nor the evaluators were influenced by the treatment condition, thereby maintaining the integrity and reliability of the outcome measures.

To further minimize potential bias, the intervention team, who administered the PMRT and Acupressure to the experimental group, did not disclose the group allocation to the patients. Similarly, the outcome assessors were not involved in patient care and were instructed not to inquire about treatment details, which ensured complete blinding throughout the study.

#### 2.5.1. TSK

The TSK assesses fear of movement/(re) injury and ranges from 17 to 68, indicating the degree of fear; higher scores denote increased apprehension. It has a Cronbach's alpha of 0.70–0.92 and a test–retest reliability above 0.80 [22]. The Chinese version of TSK was cross-culturally adapted and validated by Wei et al. [23] displaying good reliability (Cronbach's alpha: 0.74) and validity (test–retest reliability: 0.86). Data from TSK were gathered within 2 days postoperation, on the first day after intervention (i.e., the third postoperative day), the fifth day (i.e., the seventh postoperative day), and on the day of discharge.

#### 2.5.2. Visual Analogue Scale (VAS)

Pain levels were quantified using VAS, which rates pain intensity on a 100-mm line ranging from 0 (*no pain*) to 10 (*severe pain*). The VAS is widely deemed a credible and efficacious pain rating tool [24]. VAS scores were acquired during similar timeframes as the TSK.

#### 2.5.3. American Knee Society Score (KSS)

The KSS scores knee functionality in two domains: self-reported symptoms, signs, and a functional component reflecting walking and stair climbing abilities. Higher scores represent more optimal knee function and mobility capacities. Each KSS domain is rated from 0 to 100 and can be considered individually or collectively. This scoring was performed on the day of patient discharge.

### 2.6. Data Collection

Within 48 h postsurgery, patient demographics including gender, age, ethnicity, height, weight, marital status, type of medical insurance, education level, religious beliefs, occupation, income level, and comorbidities are gathered to establish baseline characteristics.

Before the intervention, and then on the first and fifth days following the intervention, as well as on the day of discharge, the TSK and VAS are employed to evaluate scores for kinesiophobia and pain in patients undergoing meniscectomy. On the discharge day, the KSS is used to assess the knee joint function scores.

**Figure 1:**
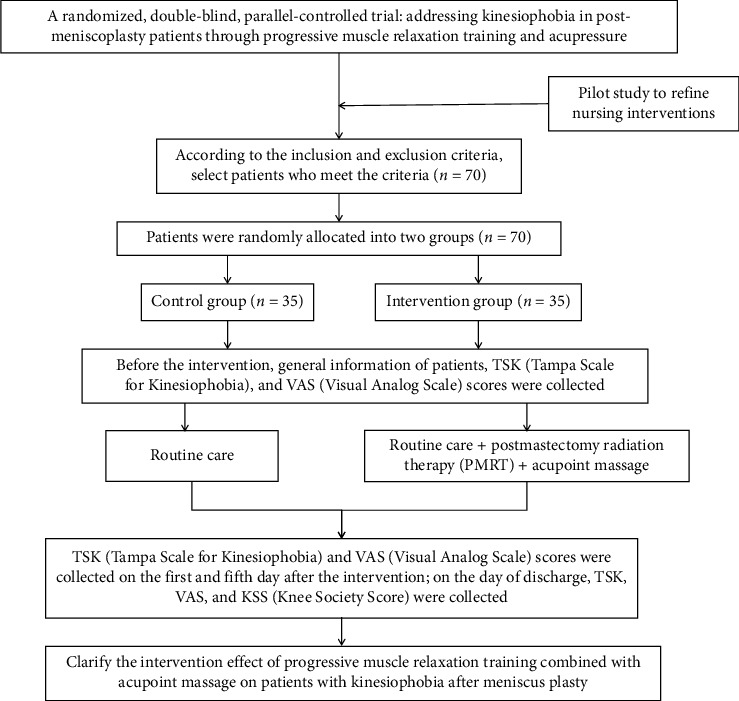
Process diagram.

The TSK and VAS questionnaires are filled out by the patients themselves. Should the patient be unable to complete the forms, for reasons such as physical incapacity or language barriers, a research team member may fill in the forms based on the patient's expressions, ensuring maximum interaction to accurately capture the patient's sentiments and observations. The KSS, assessing knee joint function, is completed by the attending physician or professional medical personnel according to observations and evaluations. The process diagram of the research procedure is shown in Figure 1.

### 2.7. Statistical Analysis

All data were assigned unique identifiers and entered into a data sheet using Excel software, with double entry and verification by two individuals. Statistical analyses were conducted using SPSS software Version 26.0. Descriptive statistics such as means, standard deviations, frequencies, and percentages were used to summarize patient characteristics.

Measurement data that were normally distributed and homoscedastic were compared using analysis of variance (ANOVA) and *t*-tests, while nonparametric tests (e.g., Mann–Whitney *U* test) were applied to data not meeting normality assumptions. Categorical data were analyzed using the chi-square test. A *p* value of less than 0.05 was considered statistically significant.

Efforts were made to prevent missingness through patient reminders, flexible scheduling, and research staff assistance. For isolated missing data points, the last observation carried forward (LOCF) method was applied. When missing data exceeded 5%, multiple imputation (MI) with five iterations was used, incorporating baseline variables to improve estimation accuracy. Intention-to-treat (ITT) and per-protocol analyses were performed, with sensitivity analyses ensuring that conclusions remained robust across different imputation methods.

## 3. Results

### 3.1. Completion of Cases

This study included 70 patients with kinesiophobia following meniscectomy, divided into a routine care group of 35 patients and an experimental group of 35 patients. Out of the 70 enrolled participants, 65 completed the trial.

During the study, 2 patients from the control group and 3 from the experimental group were discharged due to the epidemic, resulting in incomplete data collection; thus, these patients were excluded. The treatment duration for all excluded patients was less than two-thirds of the expected course, and therefore they were not considered in the subsequent data analysis. The final sample comprised 33 patients in the control group and 32 in the experimental group, totaling 65 patients. The dropout rate was approximately 7.14%, which is within the statistically acceptable range.

### 3.2. Baseline Characteristics Comparison Between the Two Groups

The sociodemographic data of both groups were compared, with gender, ethnicity, marital status, religious beliefs, type of medical insurance, and occupation analyzed using the chi-square test. Age and body mass index (BMI), which were measurement data adhering to a normal distribution and homogeneity of variance, were compared using the *t*-test. Education level and comorbidities, considered as ordinal data, were compared using rank-sum tests. There were no significant differences found between the general characteristics of the two groups (*p* > 0.05), indicating comparability in sociodemographic data between the groups. Detailed information is provided in Table 1.

### 3.3. Postintervention Kinesiophobia Score Outcomes Between Groups

TSK scores at various time points (within 48 h after surgery, on the first day after the intervention, on the fifth day after the intervention, and on the day of discharge) were compared between the two groups of patients. A repeated-measures ANOVA was utilized due to the data meeting the assumptions of normal distribution and homogeneity of variances. Mauchly's test indicated that the assumption of sphericity had been violated (*p* < 0.001), and thus, a multivariate variance analysis was performed. According to the results presented in Table 2, there was a significant time effect (*F*_time_ = 421.31, *p* < 0.001) and a significant between-group effect (*F*_between−group_ = 5.74, *p* < 0.001), indicating differences in TSK scores between the groups. Furthermore, a significant interaction effect was observed (*F*_interaction_ = 3.83, *p* < 0.001), meaning there was an interaction between the intervention and time.

**Figure 2:**
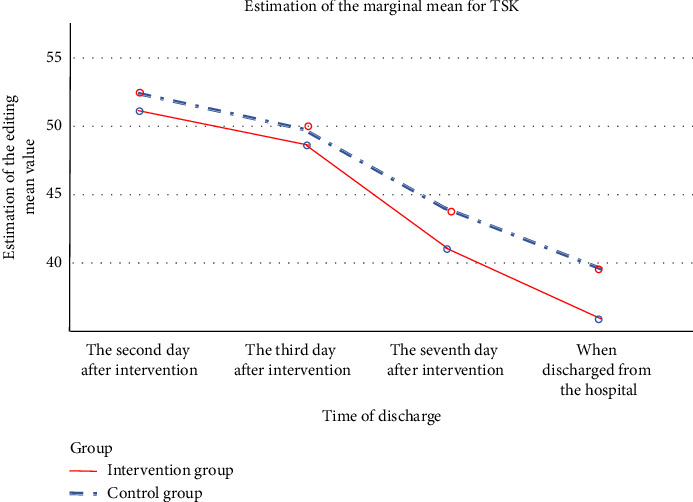
Trend of phobia scores in both groups.

The data from Table 2 show that the preintervention TSK scores (within 48 h after surgery) for the experimental and control groups were (51.00 ± 3.35) and (52.12 ± 3.92) points, respectively. An independent samples *t*-test revealed no statistically significant difference between these scores (*p* = 0.220 > 0.05), suggesting that baseline kinesiophobia levels were comparable between the two groups. On the first day postintervention, the TSK scores for the experimental group were (48.88 ± 3.33) points, and (49.73 ± 3.61) points for the control group, with no statistically significant difference in the results between the groups (*p* = 0.326 > 0.05). However, on the fifth day postintervention and on the day of discharge, TSK scores in the experimental group were lower than those in the control group, with significant statistical differences observed between the groups' TSK scores (*p* < 0.05). The multivariate ANOVA results indicated significant effects for time, between-group, and interaction. The significant time effect indicated substantial differences in TSK scores across different time points (*F*_time_ = 421.31, *p* < 0.001). The significant between-group effect showed differences in TSK scores between the experimental and control groups (*F*_between−group_ = 5.74, *p* < 0.001). The significant interaction effect demonstrated an interaction between the intervention and time (*F*_interaction_ = 3.83, *p* < 0.001). For further details, see Table 2 and Figure 2.

### 3.4. Postintervention Pain Scores Comparison Between Groups

**Figure 3:**
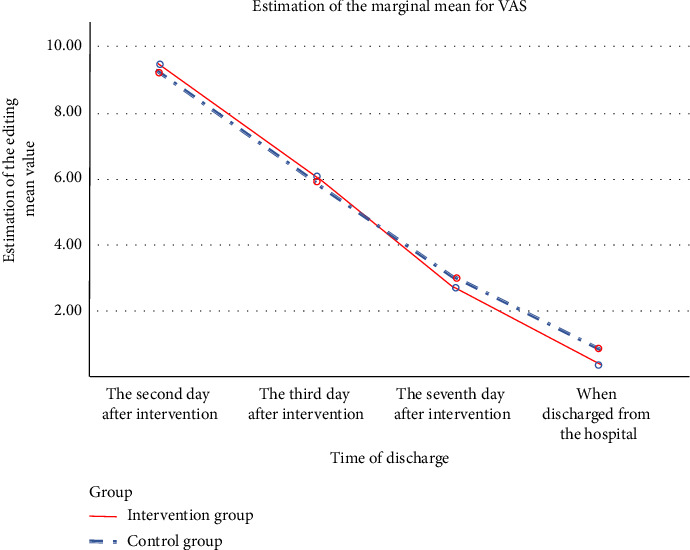
Trends in pain scores in both groups.

As indicated by the data in Table 3 prior to intervention (within 48 h postsurgery), the VAS scores for the experimental and control groups were (9.28 ± 0.73) and (9.18 ± 0.81) points, respectively. An independent samples *t*-test showed no statistically significant difference (*p* = 0.605 > 0.05), suggesting baseline comparability between the groups regarding VAS scores. As the intervention proceeded, a decreasing trend in VAS scores was noted in both groups. Postintervention VAS scores of the experimental group were (7.47 ± 1.22) points, and those of the control group were (7.36 ± 1.27) points. The difference between the groups was not statistically significant (*p* = 0.735 > 0.05). However, on the fifth day following intervention and on the day of discharge, the experimental group's VAS scores were lower than those of the control group, with a statistically significant difference between the groups (*p*  < 0.05). Results from the multivariate ANOVA revealed significant time, between-group, and interaction effects. The significance of the time effect confirmed a significant change in VAS scores over time (*F*_time_ = 1654.705, *p* < 0.001). The significance of the between-group effect indicated differences in VAS scores between the experimental and control groups (*F*_between−group_ = 4.214, *p* < 0.001). The significance of the interaction effect suggested an interactive influence between the intervention and time (*F*_interaction_ = 11.213, *p* < 0.001). Refer to Table 3 and Figure 3 for details.

### 3.5. Comparison of Postintervention Knee Joint Function Scores Between Groups

The results for the knee joint scores were (63.94 ± 7.56) points for the experimental group and (59.88 ± 5.89) points for the control group. Analysis using an independent samples *t*-test revealed a significant difference between the two groups (*t* = 2.42, *p* = 0.019 < 0.05), signifying a statistically meaningful outcome. Furthermore, in terms of knee joint function scores, the experimental group scored (51.72 ± 8.20) points, while the control group scored (46.52 ± 7.95) points. Here again, the difference in knee joint function scores between the two groups was statistically significant (*t* = 2.60, *p* = 0.012 < 0.05). Therefore, the experimental group exhibited statistically superior results in both knee joint scores and knee joint function scores when compared to the control group. Refer to Table 4 for details.

## 4. Discussion

As the population ages and awareness of fitness increases, the incidence of meniscal injuries has been steadily climbing. Such injuries hinder the joint from fully extending, thereby limiting normal activity. If meniscal injuries are not addressed in a timely manner, inflammatory responses in the joint may worsen, leading to severe knee pain and functional impairment that significantly disrupt daily life. Additionally, without intervention, the functionality of joint tissues could be compromised, potentially advancing to more severe osteoarthritis, posing a grave threat to the patient's health [25, 26].

The average age of subjects included in this study was (67.03 ± 8.26) years, with a predominant representation of the middle-aged and elderly demographic. This is in alignment with the studies conducted by Hohmann [1], correlating with the intensifying population aging and the rising incidence of degenerative knee joint diseases. Age also has been shown to positively correlate with the occurrence of postoperative kinesiophobia, indicating that the incidence is higher among older patients [27].

The subjects in this study had an average BMI of (25.09 ± 2.88) kg/cm^2^, placing them generally in the overweight category or above. This finding confirms that BMI is a risk factor for musculoskeletal diseases, consistent with research by Xu et al. [28]. Studies by Mellion and Grover [29] further highlight the close relationship between obesity and knee joint diseases, with higher BMI indices exerting greater strain on knee joints and increasing susceptibility to cartilage damage.

A greater number of female subjects were included in this study than males. Several factors may contribute to this disparity: Firstly, women have a lower pain threshold and less tolerance to pain. Research by Grosu, Lavand'Homme, and Thienpont [30] shows that women, as compared to men, have lower pain thresholds and tolerances, particularly notable after orthopedic surgeries. Dejour et al. [31] also discovered that the rate of postoperative kinesiophobia in female patients undergoing total knee arthroplasty was 1.85 times that of males. Secondly, as pointed out by Sharma, Tiwari, and Dixit [32], the incidence of musculoskeletal diseases increases rapidly among individuals aged 50–70, with postmenopausal women facing a higher risk than men of the same age, possibly related to decreased estrogen levels following menopause [33]. Consequently, middle-aged and elderly female patients with musculoskeletal conditions are more likely to experience kinesiophobia after surgery, a correlation that was also verified in this study.

In the present study, preintervention scores for kinesiophobia averaged (51.00 ± 3.35) in the experimental group and (52.12 ± 3.92) in the control group, with no significant statistical difference between the two (*p* > 0.05), indicating comparability in the level of fear of movement among patients post-meniscectomy at baseline. Both groups exhibited relatively high kinesiophobia scores postoperatively, which may primarily be attributed to significant pain experienced after orthopedic surgery. Concerns that early movement might aggravate pain or cause reinjury may lead patients to adopt a belief that immobility equates to pain relief. On the first day following intervention, the experimental group's average score for kinesiophobia was (48.88 ± 3.33) and the control group's was (49.73 ± 3.61). Although scores decreased from preintervention levels, they remained high, with no statistically significant difference between the groups (*p* > 0.05). This can be mainly ascribed to the brief duration of the intervention, resulting in less pronounced effects. However, by the fifth day postintervention and on the day of discharge, the experimental group's kinesiophobia scores were lower than those of the control group, and the difference was statistically significant (*p* < 0.05). As shown in Table 2 and Figure 2, the level of postoperative kinesiophobia diminished over time, with the experimental group consistently scoring lower than the control group from the first day postintervention through to the day of discharge. In particular, the most rapid decline in kinesiophobia scores occurred between the first and fifth days. There was a significant difference in scores between the experimental and control groups by the fifth day postintervention and the day of discharge, consistent with the findings of the study by Cici *R* [34], suggesting that a combination of PMRT and acupressure intervention can effectively reduce patients' level of kinesiophobia.

The study's results revealed that, prior to intervention, the baseline knee joint pain intensity scores for the experimental and control groups were (9.28 ± 0.73) and (9.18 ± 0.81), respectively, with no statistical difference, indicating the comparability of postmeniscectomy knee joint pain among patients. Postoperative knee pain levels were high in both groups, likely due to cartilage trauma and postoperative joint swelling [35]. Other studies also recognized high BMI and advanced age as risk factors for post-meniscectomy pain [36]. On the first day postintervention, the average knee pain intensity score decreased to (7.47 ± 1.22) in the experimental group and (7.36 ± 1.27) in the control group, indicating a reduction but still moderate levels of pain with no significant difference between groups. This primarily stems from the short duration of the intervention, which was insufficient to produce a marked effect. However, pain intensity in the experimental group was significantly lower than the control group on the fifth day postintervention and on the day of discharge. As noted from Table 3 and Figure 3, the intensity of knee joint pain following meniscectomy decreased over time. From the first postintervention day until discharge, the pain intensity scores for the experimental group were consistently lower than those of the control group, particularly during the period from the first to the fifth day. There was a significant difference in pain scores between the experimental and control groups on the fifth day postintervention and the day of discharge. These findings were closely aligned with those of Shaoping [37], and superior to the study by Xiuxiang [38], suggesting that PMRT combined with acupressure can effectively alleviate patients' knee joint pain.

In this study, the self-assessment scores for knee joint function in the experimental group surpassed those of the control group, while the control group's scores for knee joint function were lower than those of the experimental group. On the day of discharge, both groups' self-assessment scores and function scores for knee joints were at a moderate level. A potential explanation may be that patients in this study, presenting with post-meniscectomy kinesiophobia, were of an older age with more severe meniscal degeneration. Moreover, patients in the control group had a higher BMI index, resulting in increased load on the knee joints and a subsequent decrease in the self-repair ability of articular cartilage. Additionally, the rehabilitation period from surgery to discharge was comparatively brief, necessitating continued long-term functional exercise postdischarge to restore knee joint function to a high level. Notably, the recovery of knee joint function in the experimental group post-meniscectomy was superior to that of the control group. This aligns with studies by Yueguang and Zhiyu [39, 40], further corroborating the positive effect of PMRT coupled with acupressure intervention on the recovery of knee joint function in patients with post-meniscectomy kinesiophobia.

Our study demonstrated that the combination of PMRT and acupressure significantly reduced kinesiophobia in post-meniscoplasty patients. Kinesiophobia, or the fear of movement, can lead to psychological barriers that prevent patients from fully engaging in rehabilitation, thus delaying recovery and increasing the risk of further injury. By addressing this psychological component, our intervention not only facilitated more effective physical rehabilitation but also improved patients' confidence in their ability to move without pain or injury.

The reduction in kinesiophobia through nonpharmacological methods such as progressive muscle relaxation and acupressure is particularly relevant in clinical practice [41]. These methods are cost-effective and accessible, making them suitable for integration into standard rehabilitation protocols for a variety of musculoskeletal conditions, including meniscal injuries, osteoarthritis, and other joint disorders. Moreover, the mental and emotional benefits of reducing kinesiophobia—such as enhanced patient morale, reduced anxiety, and improved quality of life—can contribute to overall better outcomes in terms of functional recovery and long-term health [42–44].

Furthermore, the feasibility of these interventions in outpatient or even home-based settings makes them highly applicable in resource-limited environments, where access to traditional rehabilitation services may be restricted. Given their simplicity, these techniques can be easily taught to patients, enabling them to manage their own rehabilitation at home, thus promoting greater patient independence and long-term adherence to rehabilitation regimens.

## 5. Limitations

Due to research time constraints and conditions, this study was conducted at a single tertiary A-grade hospital in Lanzhou, which may limit the diversity of patient samples. The findings may not be directly applicable to patients treated in secondary hospitals, rural healthcare settings, or international clinical environments with different rehabilitation protocols.

Additionally, the patient population was relatively homogeneous, consisting primarily of individuals from an urban setting. Differences in demographic factors, socioeconomic status, and cultural perceptions of pain and rehabilitation may affect the generalizability of the intervention.

Another potential limitation is selection bias, as participants voluntarily enrolled in the study. Patients who were more motivated to recover may have been more likely to participate, possibly influencing the outcomes. Future research should incorporate randomized multicenter trials with broader participant inclusion criteria to confirm the robustness of the results.

Furthermore, differences in clinical practices across healthcare institutions may impact the applicability of the intervention in other settings. While our study provides promising evidence for the use of progressive muscle relaxation and acupressure in reducing kinesiophobia, additional studies across diverse healthcare systems are necessary to validate these findings.

## 6. Conclusion

The findings from this randomized controlled trial underscore the efficacy of PMRT combined with acupressure in managing kinesiophobia among post-meniscoplasty patients. The intervention significantly reduced the levels of fear associated with movement, decreased pain, and importantly, facilitated the participation in functional exercises crucial for the recovery of knee joint function. This multifaceted approach not only addresses the physical aspects of rehabilitation but also incorporates psychological and traditional Chinese medicinal techniques to provide a comprehensive, safe, and easily implementable method in clinical settings.

## Figures and Tables

**Table 1:** Comparison of sociodemographic data between the two groups of patients (*N* = 65).

Factor		Experimental group (*n* = 32, *n* (%)/($\overset{¯}{X\;}$ ± *S*))	Control group (*n* = 33, *n* (%)/($\overset{¯}{X}$ ± *S*))	*Z/*χ^2^*/t*	*p*
Age (years)		68.08 ± 8.23	66.03 ± 8.29	0.99	0.325

BMI (kg/cm^2^)		25.15 ± 2.27	25.04 ± 3.40	0.15	0.878

Sex	Man	11 (16.90%)	11 (16.90%)	0.01^a^	0.929
	Woman	21 (32.40%)	22 (33.80%)		

Marital status	Married	28 (43.10%)	30 (46.20%)	1.96^a^	0.966
	Other	4 (6.20%)	3 (4.60%)		

Nation	The Han nationality	28 (43.10%)	28 (43.10%)	0.00^a^	1.000
	The Hui nationality	3 (4.60%)	3 (4.60%)		
	Other	1 (1.50%)	2 (3.10%)		

Educational status	Primary school and illiteracy	6 (9.20%)	5 (7.70%)	−0.04^b^	0.967
	Junior middle school	10 (15.40%)	12 (18.50%)		
	Senior middle school	11 (16.90%)	11 (16.90%)		
	College degree or above	5 (7.70%)	5 (7.70%)		

Religion	Have	6 (9.20%)	5 (7.70%)	0.15^a^	0.699
	No	26 (40.00%)	28 (43.10%)		

Medical insurance	New rural cooperative	12 (18.50%)	14 (21.50%)	0.46^a^	0.795
	Urban health care	18 (27.7%)	16 (24.60%)		
	At one's own expense	2 (3.10%)	3 (4.60%)		

Occupation	Farmer	7 (10.90%)	6 (9.40%)	0.98^a^	0.613
	Staff and workers	15 (23.40%)	20 (31.30%)		
	Other	9 (14.10%)	7 (10.90%)		

Combined with diseases (species)	No	3 (4.60%)	3 (4.60%)	−0.56^b^	0.577
	One type	8 (12.30%)	6 (9.20%)		
	Two or more	21 (32.30%)	24 (36.90%)		

^a^The statistical method using chi-square test.

^b^The statistical method using rank sum test.

**Table 2:** Comparison of TSK score results between the two patients at different time points ($\overset{¯}{X}$* ± S*).

Group	Before the intervention (within 48 h after the surgery)	The one day after intervention	The five day after intervention	On the day of discharge	*F*_time_	*F*_between−group_	*F*_interaction_
Experimental group	51.00 ± 3.35	48.88 ± 3.33	41.25 ± 2.91	36.97 ± 2.38	421.308⁣^∗∗^	5.744⁣^∗∗^	3.833⁣^∗∗^
Control group	52.12 ± 3.92	49.73 ± 3.61	43.70 ± 3.27	39.45 ± 2.58			
*T*	−1.24	−0.99	−3.18	−4.04			
*p*	0.220	0.326	0.002	< 0.001			

⁣^∗∗^*p* < 0.001.

**Table 3:** Comparison of pain scores at different time nodes in the two groups ($\overset{¯}{X}$ ± *S*).

Group	Before the intervention (within 48 h after the surgery)	The one day after intervention	The five day after intervention	On the day of discharge	*F*_time_	*F*_between−group_	*F*_interaction_
Experimental group	9.28 ± 0.73	7.47 ± 1.22	3.69 ± 0.78	1.75 ± 0.67	1654.705⁣^∗∗^	4.214⁣^∗∗^	11.213⁣^∗∗^
Control group	9.18 ± 0.81	7.36 ± 1.27	4.79 ± 1.24	2.48 ± 0.94			
*T*	0.52	0.34	−4.26	−3.62			
*p*	0.605	0.735	< 0.001	< 0.001			

⁣^∗∗^*p* < 0.001.

**Table 4:** Comparison of the two groups ($\overset{¯}{X}$ ± *S*).

Group	*n*	Knee score	Knee joint function score
Experimental group	32	63.94 ± 7.56	51.72 ± 8.20
Control group	33	59.88 ± 5.89	46.52 ± 7.95
*T*		2.42	2.60
*p*		0.019	0.012

## Data Availability

Data are available by contacting the corresponding authors (R.X. and Y.G.) through e-mail.

## Appendix 1: Specific Content of Intervention

The specific contents of postoperative routine care are as follows:

1. Vital signs monitoring: Continuous electrocardiographic monitoring is carried out during the postanesthesia recovery period, closely monitoring the patient's temperature, pulse, respiration, and blood pressure; once the condition stabilizes, vital signs are monitored at regular intervals and records are maintained.
2. Positioning care: Within 6 h postsurgery, the patient should lie flat without a pillow, the affected limb should be kept in an extended knee position, and a soft pillow should be placed under the heel to elevate the knee joint by 15–20 cm to promote blood return.
3. Nutritional care: Explain and guide the patient on the required dietary type, following the principle from liquid diet to semi-liquid diet to regular food, and change the diet timely according to the specific condition of the patient.
4. Pain management: Guide the patient in the correct use of analgesic medications, explain to the patient and their family the principles of analgesia and the mechanisms involved, as well as precautions when using analgesic drugs, and teach the patient to use a VAS for pain to accurately express their pain level.
5. Psychological care: Enhance rounds, encourage patients to actively express their psychological concerns, understand the patient's psychological state. Explain the disease-related rehabilitation knowledge such as postoperative functional exercises, assess and adjust the activity plan timely according to the patient's condition, alleviate negative emotions such as anxiety, irritability, and confusion, and help patients build confidence in their postoperative recovery.
6. Functional exercise: Distribute rehabilitation exercise manuals postoperatively, and guide the patients in functional exercises through videos and live demonstrations, encouraging them to perform early knee joint exercises, such as prolonged contraction movements of the quadriceps (5–10 times/set, 3 sets/day), and ankle pump exercises (10–20 times/set, 3 sets/day).
7. Health education: Inform patients that early postoperative functional exercises can reduce complications such as postoperative adhesions and deep vein thrombosis of the lower limbs, explain the dangers of kinesiophobia to the patients, guide them to overcome the fear of movement due to pain, help them understand the importance of early functional exercises, and enhance their confidence in postoperative rehabilitation training.

The specific steps of PMRT are as follows.

1. Preparation before relaxation: The environment should be quiet, with soft lighting and suitable temperature. The researcher explains to the patient the training objectives, basic methods, duration, and precautions of PMRT. Before the start of PMRT, the patient is reminded to use the restroom, and the patient then lies down in a supine or semi-recumbent position, with eyes gently closed, legs apart, and hands placed on the abdomen or at the sides of the body. The patient is informed to concentrate their attention in the most comfortable posture.
2. Prerelaxation training: The researcher first guides the patient to take deep breaths, tense the hand muscles for about 10–15 s, and then slowly relax the muscles for about 10–15 s, allowing the patient to feel the difference between muscle tension and relaxation. Muscle relaxation starts with the hands, typically beginning with the right hand—first making a fist, then squeezing it tightly, then even more tightly, and finally releasing suddenly to experience the sensation of tension followed by relaxation. The process continues in the following order: right forearm and biceps, left hand, left forearm and biceps, forehead, eyes, mouth, shoulders, neck, back, chest, abdomen, buttocks, thighs, calves (toes up, toes down), and both feet (inward and outward).
3. Conducting PMRT training: The researcher measures the patient's baseline pulse and guides the experimental group to follow the PMRT course published by the Chinese Medical Association Audiovisual Publishing House on mobile. The relaxation routine is as follows:
  Step 1: “Deep breath and hold for a while.” Pause for 10 s “Okay, please breathe out slowly, breathe out slowly.” Pause for 5 s “Now let's do it again.” Repeat as before.
  Step 2: “Now, extend your forearm, clench your fist, grip your fist tightly and feel the tension in your hand.” Pause for 10 s “Okay, please relax, try to relax your hand, and experience the feeling of relaxation. You might feel heavy, light, and warm. These are sensations of relaxation, please feel them.” Pause for 5 s “Let's do it again now.” Repeat as above.
  Further steps continue in a similar manner, focusing progressively on different muscle groups and incorporating sessions of tension and relaxation.
4. After PMRT: The researcher measures the patient's pulse again and observes the patient's state. Training success criteria include: a reduced pulse rate; the patient's facial expression is calm, breathing is slow, and muscles in all parts of the body are relaxed, with the feet showing outward expansion when the patient is lying down.
5. During the training process, the researcher guides the patient according to the PMRT training course on the mobile phone, ensuring that the relaxation movements and breathing actions are synchronized and coordinated. This process helps the patient experience both tension and relaxation, achieving total relaxation of all body muscles.
6. Throughout the training process, the researcher closely observes the patient's reactions, promptly understanding their feelings, monitoring changes in vital signs, and related symptoms. The patient is instructed to train according to their individual condition, and if symptoms such as increased heart rate, shortness of breath, or elevated blood pressure occur during or after the training, the session should be immediately stopped to assist the patient in resting and observing their condition, and if necessary, consult a doctor.
